# Eye movement alterations in presymptomatic *C9orf72* expansion gene carriers

**DOI:** 10.1007/s00415-021-10510-z

**Published:** 2021-03-11

**Authors:** Anna Behler, Antje Knehr, Julia Finsel, Martin S. Kunz, Christina Lang, Kathrin Müller, Hans-Peter Müller, Elmar H. Pinkhardt, Albert C. Ludolph, Dorothée Lulé, Jan Kassubek

**Affiliations:** 1 Department of Neurology, University of Ulm, Oberer Eselsberg 45, 89081 Ulm, Germany; 2 Neuropsychology, Department of Neurology, University of Ulm, Ulm, Germany

**Keywords:** Amyotrophic lateral sclerosis, *C9orf72*, Saccades, Cognition, Presymptomatic gene carriers

## Abstract

**Objective:**

The clinical manifestation of amyotrophic lateral sclerosis (ALS) is characterized by motor neuron degeneration, whereas frontotemporal dementia (FTD) patients show alterations of behavior and cognition. Both share repeat expansions in *C9orf72* as the most prevalent genetic cause. Before disease-defining symptoms onset, structural and functional changes at cortical level may emerge in *C9orf72* carriers. Here, we characterized oculomotor parameters and their association to neuropsychological domains in apparently asymptomatic individuals with mutations in ALS/FTD genes.

**Patients and methods:**

Forty-eight carriers of ALS genes, without any clinical symptoms underwent video-oculographic examination, including 22 subjects with *C9orf72* mutation, 17 with *SOD1*, and 9 with other ALS associated gene mutations (*n* = 3 *KIF5A*; *n* = 3 *FUS/FUS* + *TBK1*; *n* = 1 *NEK1*; *n* = 1 *SETX*; *n* = 1 *TDP43*). A total of 17 subjects underwent a follow-up measurement. Data were compared to 54 age- and gender-matched healthy controls. Additionally, mutation carriers performed a neuropsychological assessment.

**Results:**

In comparison to controls, the presymptomatic subjects performed significantly worse in executive oculomotor tasks such as the ability to perform correct anti-saccades. A gene mutation subgroup analysis showed that dysfunctions in *C9orf72* carriers were much more pronounced than in *SOD1* carriers. The anti-saccade error rate of ALS mutation carriers was associated with cognitive deficits: this correlation was increased in subjects with *C9orf72* mutation, whereas *SOD1* carriers showed no associations.

**Conclusion:**

In *C9orf72* carriers, executive eye movement dysfunctions, especially the increased anti-saccade error rate, were associated with cognitive impairment and unrelated to time. These oculomotor impairments are in support of developmental deficits in these mutations, especially in prefrontal areas.

## Introduction

The underlying pathological process in neurodegenerative conditions likely begins some time before the emergence of clinical symptoms. Signs may be decades long in many neurodegenerative disorders (like Parkinson’s and Alzheimer’s disease), but there is still limited knowledge about the duration of the presymptomatic phase of amyotrophic lateral sclerosis (ALS) [1]. From an academic perspective, studies in the presymptomatic phase offer invaluable learning opportunities to study propagation patterns, characterize early genotype-associated signatures, and evaluate neurodevelopmental or environmental factors [2]. While ALS is primarily characterized by motor neuron degeneration, cognitive deficits are quite common [3–5], including changes in executive functions together with verbal fluency, language, social cognition, and memory. These deficits could be associated with white matter changes in frontal areas in ALS patients [5, 6]. Recently, alterations in the executive function of verbal fluency associated with a loss of structural integrity were observed in presymptomatic *C9orf72* carriers [7], i.e., in subjects with a hexanucleotide GGGGCC-repeat expansion in *C9orf72* which is the most prevalent genetic cause of ALS and frontotemporal dementia (FTD) in Caucasian populations [8]. Given that the *C9orf72* protein is believed to play a key role in the development of the central nervous system [9], cerebro-structural and functional (e.g. neuropsychological) dysfunctions in a preclinical state support the assumption of a developmental tardiness as a general trait in *C9orf72* carriers [7]. Presymptomatic SOD1 gene carriers also showed changes in advance of symptom onset such as a reduced number of motor units, increased cortical excitability, and differences in cervical cord tissue metabolites [10].

Although a relative preservation of eye movements is recognized as a feature of ALS, eye-tracking offers an objective means to assess extramotor cerebral involvement in ALS [11]. The oculomotor parameters being most prominently affected in ALS are assigned to frontal lobe impairment [12, 13]. Similar alterations with joint occurrence of increased error rates of anti-saccades and delayed saccades have been reported for patients with FTD [14]. For oculomotor function in ALS, it has been shown that the oculomotor decline follows a sequential pattern: after the initial disruption of executive eye movement control, the pulse-generating part of the brainstem circuity for saccade generation is affected [15].

In the present study, we characterized oculomotor parameters and their association to neuropsychological domains in a cohort of apparently asymptomatic individuals who are carrying mutations in ALS/FTD causing genes and compared *C9orf72* carriers to other gene mutation carriers.

## Methods

### Subjects

Forty-eight participants from families with at least one member diagnosed with ALS according to the revised El Escorial criteria (index patient) were included. Additionally, 54 age- and sex-matched healthy controls with no evidence for family history of ALS or FTD and without any family relations to the gene carriers were included. Statistics of detailed features of all participants are summarized in Table 1. All of the index patient’s family members were contacted and invited to take part in the study. In case of interest, they underwent a series of investigations, including video-oculographic and neuropsychological assessments during the course of 2 days.

**Table 1:** Subjects demographic and clinical characterization

	ALS gene carriers *n* = 48	Healthy controls *n* = 54	*p*	*C9orf72* carriers *n* = 22	*SOD1* carriers *n* = 17	*p*
Gender/male:female	20:28	23:31	1^a^	5:17	11:6	0.12/0.16^b^
Age/years	46, (37–52), 19–76	48, (34–60), 14–77	0.45^c^	47, (39–52), 19–67	42, (36–55), 22–69	0.18^d^
ECAS total	113, (100–121), 58–129	–	–	114, (111–122), 72–129	110, (100–121), 58–128	0.35^c^
ECAS verbal memory	18, (15–20), 3–22	–	–	18, (17–19), 6–22	17, (15–20), 6–21	0.54^c^
ECAS visuospatial function	12, (12–12), 8–12	–	–	12, (11–12), 8–12	12, (12–12), 11–12	0.08^c^
ECAS language	27, (23–28), 13–28	–	–	27, (23–28), 17–28	24, (15–27), 13–28	0.052^c^
ECAS verbal fluency	20, (16–22), 10–24	–	–	20, (18–22), 10–24	19, (16–22), 10–22	0.36^c^
ECAS executive function	39, (32–43), 17–46	–	–	40, (35–43), 25–46	39, (35–42), 17–46	0.70^c^

Data shown as median (interquartile range), minimum–maximum

^a^Fisher’s exact test refers to comparison between all gene mutation carriers and healthy controls

^b^Fisher’s exact test refers to comparison between *C9orf72* carriers and healthy controls and *SOD1* carriers and healthy controls

^c^Mann–Whitney *U* test refers to comparison between all ALS gene carriers and healthy control or between *C9orf72* and *SOD1* carriers

^d^Kruskal–Wallis analysis of variances on ranks (ANOVA) between healthy controls, *C9orf72* carriers and *SOD1* carriers

All subjects gave written informed consent for the study protocol according to institutional guidelines, which had been approved by the Ethics Committee of Ulm University, Germany (reference no. 68/19).

None of the subjects had a history or clinical manifestations of any neurological disorder, including ALS and/or FTD. All 48 subjects were tested positive for the most common ALS genes, i.e., 22 carried hexanucleotide repeat expansion mutations in the *C9orf72* gene and 17 subjects carried *SOD1* mutations; the remaining nine subjects had mutations in rare ALS genes (*n* = 3 *KIF5A*; *n* = 3 *FUS/FUS* + *TBK1*; *n* = 1 *NEK1*; *n* = 1 *SETX*; *n* = 1 *TDP43*). The genetic analysis was performed according to a state-of-the-art-protocol [16]. The asymptomatic participants were not informed about their genotypes but had received genetic counselling and knew that they might be at a risk of being a mutation carrier and at risk of developing ALS or FTD later.

### Neuropsychological assessment

For cognitive testing and to exclude clinical evidence of FTD, the German version of the ECAS [17], encompassing ALS-specific (language, verbal fluency and executive functions) and non-ALS-specific tasks (verbal memory, visuospatial abilities), was used with cut-off scores specific to cultural context [18]. The ECAS was performed by all gene carriers and controls. The maximum total score is 136, falling with cognitive decline.

### Recording of eye movement

For eye movement recording, the video-oculography device EyeSeeCam^®^ (EyeSeeTec GmbH, Fürstenfeldbruck, Germany) was used. The measurements were acquired in our oculomotor laboratory as previously described [15, 19]. All participants were comfortably seated with their eyes facing a white hemi-cylindrical screen (eyes-to-screen distance of approximately 150 cm) in a softly lit and acoustically shielded environment. To minimize confounding head motion, subjects’ heads were stabilized by an adjustable chin rest.

Smooth pursuit eye movements (SPEM) were tested in horizontal direction by a red laser spot moving sinusoidally at *f* = 0.375 Hz (range ± 20°, 12 cycles = 32 s). Subjects were instructed to track the target as accurately as possible [19]. Visually guided reactive saccades were pseudo-randomly elicited by lighting red light emission diodes so that each target step proceeded with the previous step (horizontal: 32 target steps, i.e., three times of ± 5, ± 10°, ± 15°, ± 40° and 4 times of ± 20°, targets within range ± 20°, 92.8 s acquisition time) and in a vertical direction (36 target steps, i.e., 4 times of ± 5°, ± 10°, ± 15°, ± 30° and two times ± 20°, targets within range ± 15°, 93.6 s acquisition time). The targets were presented for 2.9 s on average (range 2.1–3.5 s) in a horizontal and for 2.6 s (2.1–3.5 s) in a vertical direction. Subjects were asked to re-fixate to the new target as quickly and accurately as possible and to withhold their gaze shift until the next target appeared [20]. Performance difficulties in fixation may be ascribed to deficits of executive control, which was also tested in the following three tasks [15, 21]. Delayed saccades were tested by pseudo-randomly presenting a new red additive target at 5, 10, 20, and 40° horizontal positions (8 trials to the left and right each) after 1.7 s on average (range 1.1–2.3 s) so that each target step proceeded with the previous step. Subjects were asked to withhold their reaction to the new additive target until an acoustic ‘go’ cue was given. The cue was pseudo-randomly presented acoustically after the new additive target onset [19, 20]. Anti-saccades were tested by pseudo-randomly presenting a green target, twice for both directions, at ± 5, ± 10, ± 15, and ± 20° eccentric horizontal positions after 2.6 s on average (range 2.1–3.0 s). Participants were requested to instantly initiate a gaze shift towards the mirror (opposite) position of the new target. A practice training session of five runs with different eccentricities was administered before the anti-saccade and delayed saccade tasks. Rapid alternating voluntary gaze shifts were evoked in horizontal and vertical directions by requesting subjects to saccade for 30 s as rapidly as possible, back and forth between two steady green targets arranged symmetrically about the primary direction with 20° horizontal or vertical angular separation.

### Analysis of eye movement recordings

The interactive MATLAB^®^ (The Mathworks Inc., Natick, MA, USA)-based in-house software package OculoMotor Analysis was used for analysis of eye movement recordings according to a data processing pipeline as previously described in detail [15, 21, 22]. Neither the patient group nor the control group exhibited systematic differences between the right and the left eye so that the binocular recording was merged averaging the monocular recordings [22]. All measurements were visually inspected for quality assurance. Smooth pursuit eye movement yielded the smooth pursuit gain as the ratio of smooth eye velocity to target velocity [15, 19, 22]. Visually guided reactive saccades (VGRS) were characterized by the primary saccade gain, peak eye velocity (each for horizontal, up, down), and the latency (horizontal, vertical) [22, 23]. Saccadic intrusions were examined for horizontal VGRS and computed as the accumulated amplitude of saccades excluding the primary saccades and amplitudes < 2° divided by the considered time interval (i.e., ‘prevalence’ or rate of saccadic intrusions in degrees per second) [21]. For delayed saccades and anti-saccades, the percentage of errors, i.e., saccades before cue and pro-saccades, were obtained as described previously [22]. Rapid alternating voluntary gaze shifts exceeding 10° saccade amplitude were counted for the horizontal and vertical direction. The number of those shifts was arithmetically averaged for both directions since the outcomes were considered to be basically similar [22, 24]. Oculomotor parameters which correlated with age in healthy controls were adjusted for age using a linear least square fit through the results of the controls.

### Statistical analysis

The MATLAB^®^-based Statistics Toolbox was used for statistical data analyses of subject characteristics and eye movement parameters [15, 21]. We cannot assume a normal distribution of the oculomotor parameters so that we used non-parametric interference statistics to compare the eye movement parameters between the cohorts in accordance with previous studies [13, 21]. All statistical tests were 2-sided, and *p* < 0.05 was considered statistically significant.

Statistical interference between groups was analyzed using Fisher’s exact test for categorical variables or Wilcoxon–Mann–Whitney *U* test and Kruskal–Wallis analysis of variances on ranks for continuous variables, respectively. In case of three groups (*C9orf72* mutation carriers, *SOD1* mutation carriers, healthy controls), the Kruskal–Wallis analysis of variances on ranks was followed in the event of significance by Wilcoxon–Mann–Whitney *U* test. Possible relationships between eye movement parameters and clinical parameters were studied using a non-parametric Spearman rank–order correlation coefficient. The resulting *p* values were corrected for multiple comparisons using family-wise error correction.

### Longitudinal analysis

A subgroup of *n* = 17 subjects, consisting of ten *C9orf72* gene carriers, five *SOD1* gene carriers, and two with rare gene mutations, was measured a second time after 38 ± 13 months. In comparison to those who did not receive a second testing, there was no statistically significant difference regarding to age, gender, and ECAS scores. All these subjects received the same neuropsychological and video-oculographic protocol at both time points.

## Results

Eye movement control was different between the gene mutation carriers (*n* = 48) compared to healthy controls (*n* = 54) and between the subgroups *C9orf72* (*n* = 22) and *SOD1* carriers (*n* = 17) compared to healthy controls, respectively (Table 2). Specifically, gene mutation carriers performed in the delayed and anti-saccades tasks with an altered error rate by having difficulties in suppression of unwanted gaze shifts and frequently moving their eyes towards the target (pro-saccade or gaze shift before acoustic cue) (*p* < 0.01). Many of these errors were immediately corrected, indicating that the subjects had no difficulties in understanding the tasks. The performance of self-initiated gaze shifts revealed no statistical difference between gene carriers and healthy controls. While awaiting a new target position during VGRS, ALS gene carriers showed abnormally large and frequent saccadic intrusions in comparison to controls (*p* < 0.01). SPEM gain was similar to those of controls. Gain of reactive primary saccades in horizontal and downwards directions revealed no statistical difference, however, the VGRS gain in upward direction was significantly lower in comparison to controls (*p* < 0.05). Peak eye velocities of reactive primary saccades and latencies were normal.

**Table 2:** Video-oculographic parameters of mutation carriers and healthy controls

	ALS gene carriers	healthy controls	*p*^a^	*C9orf72* carriers	*SOD1* carriers	ANOVA *p*^b^
Anti-saccades error rate^c^/%	21, (13–41), 0–100	15, (6–30),0–63	**0.0091**	24, (12–49), 4–100^#^	19, (12–36), 0–87	**0.0254**
Delayed saccades errror rate^d^/%	12, (5–18), 0–91	4, (2–11), 0–21	**0.0013**	9, (5–19), 0–91^#^	14, (6–16), 0–68^#^	**0.0035**
Number of voluntary gaze shifts^e^	55, (42–64), 14–114	56, (50–64), 40–98	0.116	55, (46–61), 21–114	51, (42–68), 14–87	0.2861
Intrusion rate^f^/°/s	3.7, (2,6–6,3), 1.7–14.9	2.9, (2.2–4.5), 1.0–8.7	**0.0039**	4.9, (2.8–7.2), 1.7–14.9^#^	3.1, (2.6–5.6), 2.4–11.7	**0.0008**
SPEM gain/%	90, (76–95), 15–100	91, (77–97), 22–111	0.8196	93, (71–96), 31–100	90, (81–95), 15–100	0.8316
VGRS (horiz.) gain^g^/%	89, (84–91), 69–95	89, (86–93), 69–97	0.1871	86, (83–90), 69–95^#^	91, (89–92), 80–94	0.0652
VGRS (down) gain^g^/%	93, (89–97), 81–112	91, (86–96), 76–110	0.1999	90, (88–97), 81–104	94, (91–97), 81–112	0.4842
VGRS (up) gain^g^/%	75, (66–84), 52–92	80, (77–84), 62–96	**0.0220**	71, (65–83), 52–92^#^	76, (72–85), 57–92	**0.0070**
VGRS (horiz.) velocity^h^/°/s	420, (393–471), 349–574	432, (401–464), 245–536	0.6556	440, (398–474), 354–574	415, (394–455), 379–524	0.8144
VGRS (down) velocity^h^/°/s	391, (329–444), 254–579	393, (349–437), 227–538	0.4159	363, (310–454), 254–579	391, (332–421), 276–462	0.5936
VGRS (up) velocity^h^/°/s	434, (376–474), 309–551	432, (405–487), 216–599	0.5730	445, (393–502), 309–550	422, (369–462) 310–529	0.5611
VGRS (horiz.) latency^i^/ms	219, (208–236), 178–304	216, (202–236), 183–413	0.7048	216, (203–235), 178–304	225, (207–239), 190–255	0.9268
VGRS (vert.) latency^i^/ms	230, (213–252), 189–355	237, (215–253), 193–371	0.8748	231, (211–252), 189–307	226, (220–252), 193–287	0.8816

Data are presented as median (interquartile range), minimum–maximum. Bold values indicate significance at *p* < 0.05. Post hoc statistical comparisons for C9orf72 carriers vs. healthy controls and SOD1 carriers vs. healthy controls which reached statistical significance are indicated as ^#^

^a^Mann–Whitney *U* test between healthy controls and ALS gene carriers

^b^Kruskal–Wallis analysis of variances of ranks (ANOVA) between healthy controls, subjects with *C9orf72* mutation and *SOD1* mutation

^c^Erroneous responses (pro-saccades)

^d^Erroneous responses (saccades before cue)

^e^Saccades > 10° counted within 30 s

^f^Saccadic intrusions > 2° excluding the primary saccade, computed as the sum of saccades within VGRS acquisition time

^g^Gain of VGRS aimed at targets of 20° eccentricity obtained by linear fitting saccade amplitudes as a function of target steps

^h^Peak eye velocity of VGRS aimed at targets of 20° eccentricity obtained by non-linear interpolation along the main sequence

^i^Latencies of VGRS with respect to primary saccade onset

### Subgroup analysis of eye movement control

**Fig. 1:**
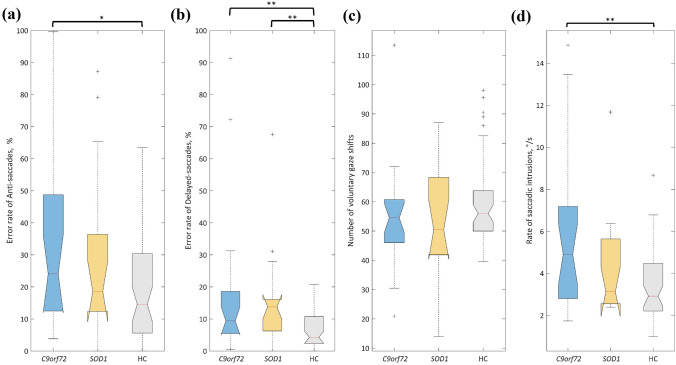
Group comparison of (**a**) the anti-saccade error rate, **b** the delayed saccade error rate, **c** the number of voluntary gaze shifts and **d** the rate of saccadic intrusions for C9orf72 carriers, SOD1 carriers, and healthy controls. Box plots showing the following statistical data: median, confidence interval of the median, the interquartile range, and possible outliers. Significance marked as *p < 0.05, **p < 0.01

ANOVA of *C9orf72* carriers, *SOD1* carriers, and healthy controls indicated significant differences between the three groups for anti- and delayed saccades, the saccadic intrusion rate, and VGRS gain, respectively. Post hoc comparison, shown in Fig. 1, revealed deficits for *C9orf72* carriers in those parameters in comparison to controls (anti-saccade error rate, *p* = 0.0242; delayed saccade error rate, *p* = 0.0091; saccadic intrusion rate, *p* = 0.0049). In contrast, the comparison of *SOD1* carriers with controls resulted in only one significant finding, i.e., the error rate of delayed saccades (*p* = 0.0097).

The subjects with *C9orf72* mutation showed an altered reactive saccade gain in comparison to controls, both upwards (*p* = 0.0242) and in horizontal directions (*p* = 0.0136). In the *SOD1* carriers, VGRS gain downwards were normal, likewise the VGRS gains up, down, and in horizontal directions.

### Longitudinal comparison of executive eye movement control

Due to the low sample size of follow-up measurements, the carriers of *SOD1* and rare gene mutations were excluded from longitudinal data analysis. The comparison of baseline measurements and follow-up measurements in *C9orf72* gene carriers demonstrated no significant change in any executive oculomotor domain nor in any cognitive subdomain of the ECAS (verbal fluency, language, executive function, memory, visuospatial function).

### Association of executive oculomotor functions with cognitive performance

The total ECAS score of the complete group of ALS gene carriers was associated with the anti-saccade error rate (*r* =  − 0.59, *p* = 0.004) and the number of voluntary gaze shifts (*r* = 0.46, *p* = 0.042), i. e., lower ECAS scores corresponded with more frequent errors in the anti-saccade task and a lower number of voluntary inhibited gaze shifts (Table 3). Having a detailed look into particular ECAS domains, those associations were reflected in a correlation of anti-saccade error rate (*r* =  − 0.63, *p* = 0.003) and the number of voluntary gaze shifts (*r* = 0.55, *p* = 0.024), respectively, with the executive function performance in ECAS. Although there was no correlation with the total ECAS score, the presence of saccadic intrusions in fixations periods was associated with a worse visuospatial function (*r* =  − 0.56, *p* = 0.014).

**Table 3:** Correlation coefficients *r* of executive oculomotor parameters with neuropsychological ECAS scores (total and single sections) in presymptomatic ALS gene carriers

	ALS gene carriers				*C9orf72* carriers				*SOD1* carriers			
	Anti-saccades error rate/%	Delayed saccades error rate/%	Number of voluntary gaze shifts	Intrusion rate/°/s	Anti-saccades error rate/%	Delayed saccades error rate/%	Number of voluntary gaze shifts	Intrusion rate/°/s	Anti-saccades error rate/%	Delayed saccades error rate/%	Number of voluntary gaze shifts	Intrusion rate/°/s
ECAS total	**− 0.59**	− 0.32	*0.46*	− 0.33	**− 0.74**	− 0.53	0.28	− 0.56	− 0.68	− 0.13	0.59	− 0.14
ECAS verbal memory	− 0.42	− 0.14	0.29	− 0.11	*− 0.73*	− 0.38	0.10	− 0.23	− 0.50	− 0.02	0.33	0.05
ECAS visuospatial function	− 0.36	− 0.34	0.19	*− 0.56*	− 0.35	− 0.39	0.17	− 0.63	–	0.20	0.19	− 0.20
ECAS language	− 0.29	− 0.18	0.24	− 0.15	− 0.54	− 0.40	0.21	− 0.54	-0.42	− 0.12	0.33	0.05
ECAS verbal fluency	− 0.43	− 0.22	0.35	− 0.28	− 0.42	− 0.20	0.33	− 0.41	− 0.68	− 0.30	0.45	− 0.19
ECAS executive function	**− 0.63**	− 0.37	**0.55**	− 0.42	*− 0.73*	− 0.67	0.30	− 0.62	− 0.77	− 0.07	*0.82*	− 0.30

The matrix represents the correlation of anti-saccade error rate, the error rate of delayed saccades, number of voluntary gaze shifts, and the saccadic intrusion rate with the total ECAS score and the following ECAS sections: verbal memory, visuospatial function, language, verbal fluency, and executive function. Significance: unbold, *p* > 0.05; italics, *p* < 0.05; bold, *p* < 0.01

For *C9orf72* carriers, the error rate of anti-saccades was strongly associated with the performance of the total ECAS (*r* =  − 0.74, *p* = 0.009) and executive function (*r* =  − 0.73, *p* = 0.034) similar to all ALS gene carriers’ ECAS executive function. In addition, a strong correlation between ECAS verbal memory score and anti-saccade error rate (*r* =  − 0.73, *p* = 0.0358) was shown. Other significant correlations between executive eye movement parameters and any ECAS section were not observed. In the subgroup of *SOD1* carriers, only the performance of self-initiated gaze shifts correlated with the ECAS executive function score (*r* =  − 0.82, *p* = 0.040), while the error rate of anti-saccades did not.

## Discussion

The video-oculographic data provided evidence for alterations in executive eye movement parameters in presymptomatic carriers of gene mutations for ALS. More specifically, impairment in executive oculomotor control was primarily observed in *C9orf72* repeat expansion carriers, long before any overt clinical signs of ALS or FTD pathologies known to be associated with *C9orf72* mutations. Compared to other ALS gene mutations, the repeat expansion in the *C9orf72* gene is common in both familial ALS and FTD [1]. At the time of study inclusion, it is not to be predicted whether *C9orf72* mutations carriers will develop either pathology of ALS or FTD in the future. With regard to oculomotor pathology, both pathologies seem to share common preclinical manifestations in those subjects with *C9orf72* repeat expansions: *C9orf72* carriers had a higher saccadic intrusion rate and they performed significantly worse in suppressing unwanted gaze shifts toward a new target during anti-saccade and delayed saccade tasks, all signs of impaired executive control of eye movements. These results are in line with the reported executive dysfunction in cognitive tasks at behavioral level reported for ALS patients [17, 25, 26]. Executive dysfunction is also characteristic for frontotemporal dementia which shares neuropathological, clinical, radiological, and genetic overlap with ALS [27]. ECAS, as an ALS-specific test that can be performed even in the presence of motor impairments, focuses on the assessment of executive functions. Thus, it was possible to detect correlations to the executive abilities in the oculomotor tasks.

We thus present hereby further evidence for clinical overlap in *C9orf72* mutation carriers who share executive functions also at the oculomotor level. Note that neither *C9orf72* nor *SOD1* carriers faced challenges in initiating saccades, which would rather be a sign of disturbed pontocerebellar circuits or impaired oculomotor brainstem nuclei. It should be noted that *SOD1* carriers also performed significantly worse than controls with respect to error rate in the delayed saccades task, but these data were not correlated with their ECAS performance. Taken together, the investigations of primarily executive oculomotor functions further support the notion of a prefrontal dysfunction in subjects with *C9orf72* mutation [7]. The different characteristics of *C9orf72* vs. *SOD1* gene carriers are also supported by associations between executive oculomotor parameters and cognitive performance. The performance in the anti-saccade error task significantly correlated with the cognitive performance in the ECAS in *C9orf72* gene carriers but not in *SOD1* gene carriers. Although it has not been entirely specified yet which cognitive functions are involved in the correct execution of anti-saccades, attention plays a role, as it does for verbal memory and executive functions. Most importantly, these alterations in executive functions remained unchanged over time in *C9orf72* carriers, implying rather a feature of executive dysfunction which is not a matter of cognitive decline over time but rather a general trait in itself [28].

Impaired cognitive function may be linked to early central nervous system development [29]. Recently, it was shown that presymptomatic *C9orf72* carriers showed cerebro-structural and cognitive dysfunctions unrelated to time [7]. The longitudinal assessments in a subset of subjects showed no significant differences in any executive oculomotor domain so that no change over the given time could be observed. None of the ALS gene carriers was diagnosed with autism or showed any clinical sign of autism; however, according to previous data [7], developmental tardiness might be a general trait in *C9orf72* carriers. It can be speculated that the alterations in the executive oculomotor parameters in *C9orf72* carriers, especially the anti-saccade error rate, might be indicating developmental deficits originating in early childhood. This hypothesis arises from the fact that these oculomotor functions develop in the first years of life: young children face challenges in suppressing unwanted pro-saccades in the anti-saccade task [30, 31], and only about the age of 10 there is an improvement in the ability to suppress reflexive saccades in the sense of a strong decrease of the amount of anti-saccade errors. Thus, we hereby support the assumption that *C9orf72* mutations are associated with impairments in oculomotor tasks which may indicate delayed brain development. It may thus represent a tardiness in neuronal development in *C9orf72* mutations as has been shown in synaptic regulation, excitotoxicity [32], neural development [9], and cognition at the behavioral level [28]. These changes were unrelated to time and can rather be regarded as a general trait in *C9orf72* carriers. As this pattern was not observed in *SOD1* mutation carriers, it can be regarded as specific to *C9orf72* alterations, as has been previously been implied by neuroanatomical and cellular pathway involvement [9, 29]. In addition to alterations in executive oculomotor functions, *C9orf72* carriers showed hypometric horizontal and asymmetric vertical saccades which indicate ‘genuine’ oculomotor dysfunctions, although the change of the horizontal saccade gain is commonly observed in patients with FTD [33] but not in ALS patients [12, 15] and might be regarded as another element of the *C9orf72*-associated overlap of motor neuron disease and frontotemporal pathology.

This study has a number of limitations. Due to the limited sample size of rare gene mutations, we could not include them in subgroup comparison. A limitation of the longitudinal analysis is the low number of participants, especially for follow-up. For the future, a validation of these results with a larger group would be favourable. Also, mutation carriers were only screened for cognitive performance and future work might include more intensive neuropsychological testing, in addition with more longitudinal assessments. Nevertheless, the association of executive function at the oculomotor level which was closely associated with the cognitive behavioral level very well implies a disruption of frontal involvement in *C9orf72* mutation carriers, long before any overt clinical signs of disease onset of either ALS or FTD.

So overall, we hereby present evidence for oculomotor impairments associated with *C9orf72* repeat expansions in apparently presymptomatic stages, which further support the concept of developmental delay associated with these mutations, especially in prefrontal areas. Biomarkers will play an important role in future therapeutic decisions now that *C9orf72* mutation carriers can be identified by genetic testing many decades before symptoms begin [34], given that, with the emergence of antisense oligonucleotide therapies, the characterization of presymptomatic disease burden has now gained practical relevance [35]. Like in presymptomatic Huntington’s disease [36] and pre-ataxic Machado–Joseph disease [37] individuals, the recording of eye movements in presymptomatic *C9orf72* gene carriers may have the potential as a biological marker. Thus, video-oculographic recording as an unbiased assessment of the neuropsychological condition might further prove its potential as a technical biomarker in the longitudinal work-up of the clinical condition from the presymptomatic to the symptomatic stage. According to our findings, *C9orf72* carriers will play a special role in this biomarker work-up as their oculomotor and cognitive impairments probably develop early in life already.

## Data Availability

Data are available upon reasonable request and require a formal data sharing agreement, which must include details on how the data will be stored, who will have access to the data and intended use of the data, and agreements as to the allocation of intellectual property.
